# Ion Channel Contributions to Wing Development in *Drosophila melanogaster*

**DOI:** 10.1534/g3.119.400028

**Published:** 2019-02-07

**Authors:** Laura Faith George, Sarala Joshi Pradhan, Danae Mitchell, Megan Josey, Justin Casey, Matthew T. Belus, Karlie N. Fedder, Giri Raj Dahal, Emily Anne Bates

**Affiliations:** Department of Pediatrics, University of Colorado School of Medicine; Aurora, CO, 80045

**Keywords:** Ion channels, channelopathy, bioelectricity, *Drosophilia*, wing development

## Abstract

During morphogenesis, cells communicate with each other to shape tissues and organs. Several lines of recent evidence indicate that ion channels play a key role in cellular signaling and tissue morphogenesis. However, little is known about the scope of specific ion-channel types that impinge upon developmental pathways. The *Drosophila melanogaster* wing is an excellent model in which to address this problem as wing vein patterning is acutely sensitive to changes in developmental pathways. We conducted a screen of 180 ion channels expressed in the wing using loss-of-function mutant and RNAi lines. Here we identify 44 candidates that significantly impacted development of the *Drosophila melanogaster* wing. Calcium, sodium, potassium, chloride, and ligand-gated cation channels were all identified in our screen, suggesting that a wide variety of ion channel types are important for development. Ion channels belonging to the pickpocket family, the ionotropic receptor family, and the bestrophin family were highly represented among the candidates of our screen. Seven new ion channels with human orthologs that have been implicated in human channelopathies were also identified. Many of the human orthologs of the channels identified in our screen are targets of common general anesthetics, anti-seizure and anti-hypertension drugs, as well as alcohol and nicotine. Our results confirm the importance of ion channels in morphogenesis and identify a number of ion channels that will provide the basis for future studies to understand the role of ion channels in development.

Ion channels are well known for their importance in excitable cells such as neurons and muscle cells, but there is also growing evidence that ion channels play a key role in regulating developmental signaling pathways, even in tissues that are non-excitable in adults. Evidence for the importance of ion channels in development can be found in the number of human syndromes associated with morphological defects caused by ion channel mutations. These defects commonly include craniofacial, limb, and digit dysmorphisms. For example, a gain-of-function missense mutation in *CACNA1C*, a gene encoding an L-type calcium channel, causes Timothy Syndrome (Splawski *et al.* 2004). Timothy Syndrome is associated with a high incidence of small upper jaw, thin upper lip, low-set ears, syndactyly (fusion of the digits of the hands or feet), and dental defects (Splawski *et al.* 2004). Similarly, Anderson-Tawil Syndrome, caused by mutations in the gene encoding the inwardly-rectifying potassium channel Kir2.1, leads to syndactyly and clinodactyly (curvature of the fingers or toes) as well as low-set ears, small lower jaw, cleft palate, and dental abnormalities (Plaster *et al.* 2001). Other channelopathies associated with a high incidence of morphological abnormalities include Temple-Baraitser Syndrome, caused by a gain-of-function mutation in the voltage-gated potassium channel EAG1, Birk-Barel Syndrome, caused by a mutation in the two-pore potassium channel KCNK9, and Keppen-Lubinsky syndrome, caused by disruption of the inwardly-rectifying potassium channel GIRK2 (Barel *et al.* 2008; Chong *et al.* 2015; Masotti *et al.* 2015; Simons *et al.* 2015).

While the importance of ion channels in development is becoming increasingly apparent, the mechanisms by which ion channel mutations disrupt developmental signaling pathways are not fully understood. Ion channels control the transmembrane potential (V_mem_) of cells. Cells within an organism have varying resting potentials creating a “bioelectric” pattern across tissues. This pattern is important for proliferation and migration as well as correct left-right patterning, tissue and organ patterning, and organ size (Levin *et al.* 2017; Levin 2014). Changes in this transmembrane potential pattern result in significant defects in development across multiple organisms. In planarians, changing the V_mem_ gradient can cause amputated trunks to regrow heads in place of tails, resulting in two-headed organisms (Durant *et al.* 2017). In *Xenopus laevis*, clusters of hyperpolarized cells are found at the locations of eyes during embryogenesis (Pai *et al.* 2012). Depolarization of these cells results in eye malformation while hyperpolarization of non-eye cells can induce the formation of ectopic eyes (Pai *et al.* 2012). The V_mem_ pattern has been found to be important within mammalian systems as well, leading to the proposal of a “bioelectric prepattern” dictating the formation of the face (Adams *et al.* 2016).

In *Drosophila melanogaster*, ion channels have been found to play a key role in early development. In *Drosophila* ovarian follicles during oogenesis the V_mem_ changes by developmental stage (Krüger and Bohrmann 2015; Woodruff *et al.* 1988). These changes in transmembrane potentials have been found to influence protein movement and distribution in the oocyte (Woodruff *et al.* 1988; Cole and Woodruff 2000). V_mem_ patterns were found to correspond with distribution patterns of calcium channels, sodium channels, proton pumps, and gap junctions (Krüger and Bohrmann 2015). When the gap junction Innexin 2 is inhibited during oogenesis, defects in oocyte development occur, further supporting the importance of these ion channels in early *Drosophila* development (Bohrmann and Zimmermann 2008). Later on in *Drosophila* development, proper functioning of the inwardly rectifying potassium channel Irk2 has been found to be essential for wing growth and patterning, suggesting that ion channels continue to influence development in *Drosophila* beyond oogenesis (Dahal *et al.* 2012; Dahal *et al.* 2017).

While it is becoming increasingly evident that ion channels are important for development, it is still not fully known which ion channel types contribute to developmental signaling pathways. *Drosophila melanogaster* is an excellent model in which to address this question because the *Drosophila* wing is acutely sensitive to changes in developmental pathways. Disruptions of the BMP/Dpp, Notch, Hedgehog, or Wingless/WNT signaling pathways all cause changes in wing development which are easily observed such as abnormal changes in vein patterning and abnormal wing size or shape (Blair 2007). Disruption of the *Drosophila* ortholog of the Anderson-Tawil Syndrome associated potassium channel Kir2.1 (Irk2), has been previously found to cause severe wing defects, demonstrating that *Drosophila* wing development is sensitive to ion channel disruptions (Dahal *et al.* 2017; Dahal *et al.* 2012). Disruptions of other channels that play roles in development also cause *Drosophila* wing defects, making the *Drosophila* wing a useful system in which to identify ion channels that influence morphogenesis.

In this study, we used the *Drosophila* wing as a readout to screen for ion channels that impact development. We identified 180 ion channel related genes that are expressed in the *Drosophila* wing disc and then used loss-of-function *Drosophila* mutant lines or the *UAS-GAL4*/*RNAi* system to individually disrupt or knockdown ion channels. We then examined the wing phenotypes of the adult progeny of these lines. Using this approach, we identified 44 ion channel related genes which cause wing development abnormalities when disrupted or knocked down. In the interest of conducting a broad screen, we only looked at one loss-of-function or RNAi knockdown line per ion channel. While deeper interpretation of any of the candidates identified in this screen will require further confirmation of the phenotypes by CRISPR-knockouts, rescue experiments, and other characterizations in lines with differing genetic backgrounds, the results of our screen provide a starting point for further investigation of the role of ion channels in development.

## Materials and Methods

### Fly stocks

The majority of the *Drosophila melanogaster* strains used were obtained from the Bloomington *Drosophila* Stock Center at Indiana University. We selected RNAi lines that were generated by the Transgenic RNAi project, completed in the lab of Norbert Perrimon at Harvard Medical School (Perkins *et al.* 2015). The *irk1*, *irk2*, and *irk3* RNAi lines were obtained from the Vienna Drosophila Resource Center (VDRC, www.vdrc.at) (Dietzl *et al.* 2007). Flies were raised on standard cornmeal food at 25°. The *w^1118^* strain was used as the wildtype control and *MS1096-GAL4* x *w^1118^* as the background control for *MS1096-GAL4*>*UAS-RNAi* crosses.

### Identification of ion channel library

To build a library of ion channels for screening we used the Flybase RNA-seq database (flybase.org) to compile a list of ion channels expressed in *Drosophila melanogaster* (Gramates *et al.* 2017). To specifically identify ion channels expressed in the wing we overlaid this list with a library of genes expressed in the wing discs of third instar *Drosophila* larvae (Ibrahim *et al.* 2013). We chose to only screen ion channels that had loss-of-function mutant lines or RNAi lines readily available from the Bloomington Drosophila Stock Center or the Vienna Drosophila Resource Center, leaving us with a list of 180 ion channels to screen.

### Fly crosses and wing phenotype scoring

For screening of *UAS-RNAi* strains, virgin *MS1096-GAL4* females were crossed with males from each *UAS-RNAi* strain and their progeny were scored for wing phenotypes. *MS1096-GAL4* fly wings were examined as controls for all RNAi knock down lines, and for candidates of interest identified using RNAi, the starting *UAS-RNAi* lines were also screened for wing phenotypes to control for possible background genotype impacts on wing morphology.

If homozygote mutants were viable, they were scored as homozygotes. If homozygotes were not viable, heterozygote mutants were screened directly for wing defects unless the balancer expressed *Serate* (*Ser*) or *Curly* (*Cy*) which would interfere with identification of wing defects. Mutant strains balanced with *Ser* or *Cy* marked chromosomes were crossed with *w*^1118^ virgin females, and heterozygous progeny not expressing *Ser* or *Cy* were selected for scoring.

The wings of at least 20 males and 20 females were scored under a stereo microscope for each mutant strain and *UAS-RNAi* cross. We looked for abnormalities in vein patterning, vein thickness, trichome or bristle pattern, wing size, wing shape, or other notable changes when compared to controls. If any abnormality was observed, wings were mounted on a slide and further observed under a histology microscope (Nikon, eclipse 80I).

Candidates of interest were defined differently for those identified using loss-of-function mutant lines and those identified using *MS1096 > RNAi* knockdown. Heterozygous *MS1096-GAL4* expressing flies have mild wing venation defects with variable penetrance up to 100% for males and a lower penetrance for females (averaging 10.8%). For the *MS1096 > RNAi* knockdown lines we therefore defined candidates of interest as lines in which female progeny had wing defects with a percent penetrance two standard deviations above the mean penetrance of defects in heterozygous *MS1096-GAL4* control female flies (at least 29%). We also examined the starting *UAS-RNAi* lines for wing phenotypes and only identified lines as candidates of interest if the penetrance of phenotypes was at least two standard deviations above both the starting *UAS-RNAi* line and the *MS1096-GAL4* line.

Less than 1% of *w^1118^* (WT) have visible wing defects. For mutant lines we therefore defined candidates of interest as lines with a wing defect penetrance greater than 20%. This threshold was set intentionally high for mutant lines even though wildtype flies have very low penetrance of wing defects to reduce the likelihood of including false positives among the candidates of interest.

### Data Availability

A full list of all RNAi lines screened can be found in Supplementary Table 1 and a full list of loss-of-function mutant lines screened can be found in Supplementary Table 2, with their observed phenotypes and percent penetrance. We provided the stock numbers from the Bloomington *Drosophila* Stock Center at Indiana University so that the same fly lines may be purchased and our studies can be replicated. Supplemental material available at Figshare: https://doi.org/10.25387/g3.7640345.

## Results and Discussion

To identify ion channel genes associated with morphological development, we compiled a library of ion-channel related genes expressed in the *Drosophila melanogaster* third instar wing disc (Ibrahim *et al.* 2013). We examined wings of flies that harbor loss-of-function alleles of these ion channels. When mutant alleles did not exist, we drove expression of siRNA against ion channels using *MS1096-GAL4*. *MS1094-GAL4* drives expression in the dorsal compartment of the wing pouch throughout the third instar larval stage allowing us to specifically assess the impact of knocking down an ion channel in the wing disc during development (Capdevila and Guerrero 1994; Lunde *et al.* 1998).

**Figure 1:**
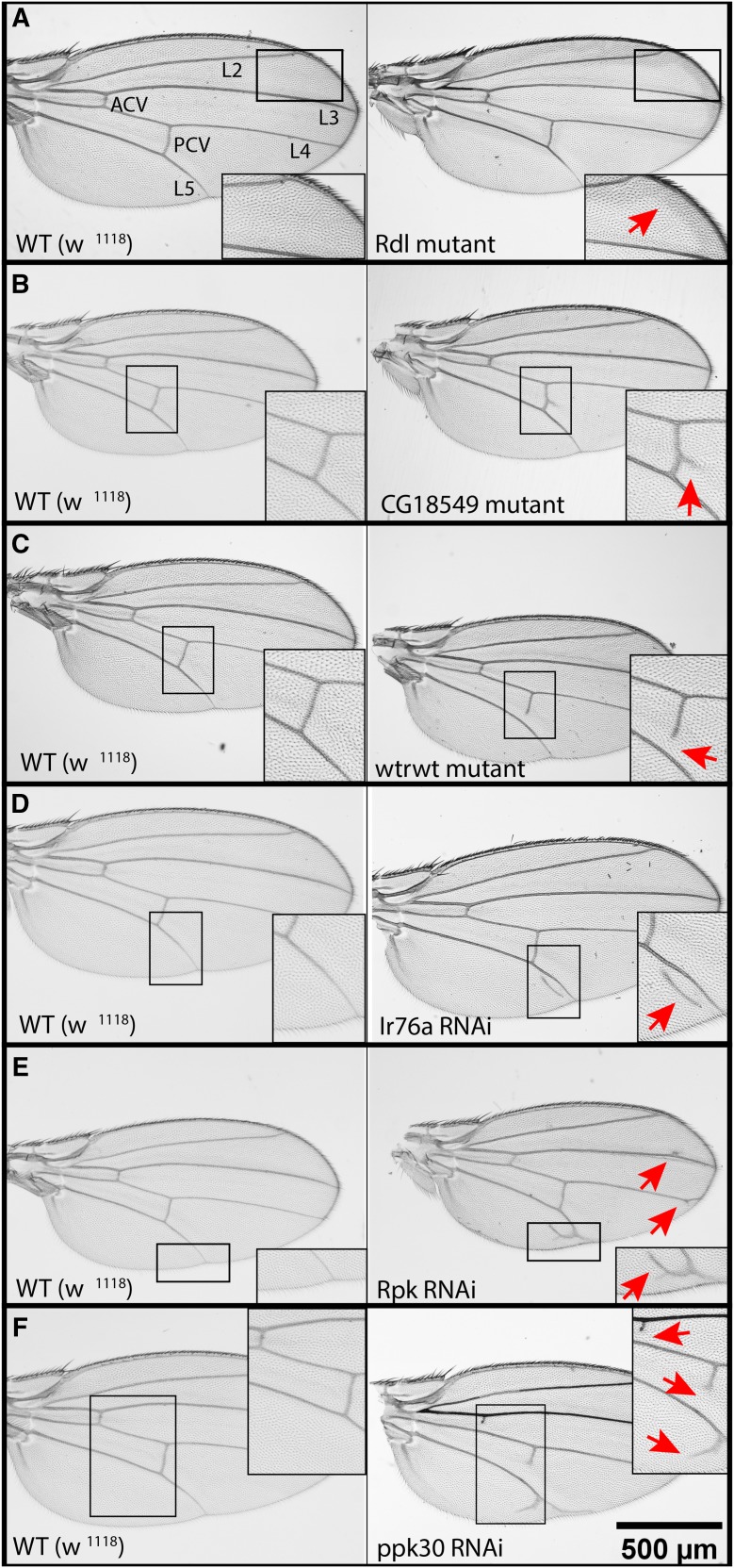
Examples of observed vein and pigment defects Disruption of 44 of the ion channel related genes screened using either loss-of-function mutations or RNAi wing-specific knockdown resulted in a wide variety of wing defects. Wild type wings have five longitudinal veins and two cross veins (A, left panel). Disruption of the 44 candidates of interest from the screen commonly resulted in abnormal wing pigment (A), posterior cross vein bifurcations (B), incomplete cross veins (C), ectopic veins (D), or longitudinal vein bifurcations (E). Some channel disruptions resulted in wings with multiple venation defects (F). The left column shows wildtype wings with matching wing sections enlarged for comparison with wing defects in right column. Arrows mark defects. The scale bar in the lower right corner represents 500 μm and applies to all panels in the figure.

**Figure 2:**
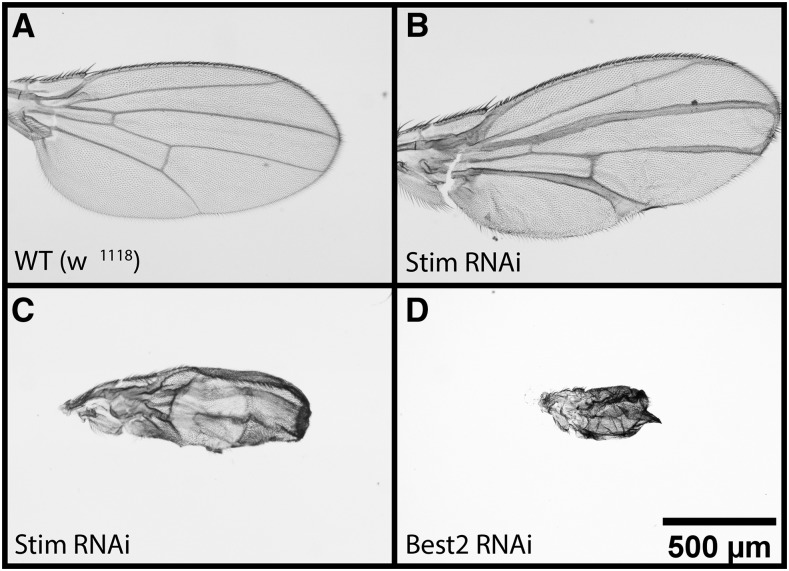
Examples of severe wing phenotypes observed Disruption of a few of the ion channels caused more severe defects. Compared to wildtype wings (A) some ion channel disruptions resulted in thickened veins (B), blistering (C), and smaller, shriveled wings (C, D). The scale bar in the lower right corner represents 500 μm and applies to all panels in the figure.

A total of 128 loss-of-function mutant lines and 61 *UAS-RNAi* lines were scored. One fourth of the ion channels screened induce significant wing phenotypes upon loss-of-function or knockdown in the wing. These phenotypes range from mild to severe, with mild defects including abnormalities in bristle patterning or wing pigmentation, incomplete wing veins, bifurcations of the wing veins, and the presence of ectopic veins (Figure 1). A few of the ion channel disruptions gave more severe wing defects including vein thickening, blistering, or complete shriveling of the wing (Figure 2).

Candidates of interest from our screen were defined as mutant lines in which more than 20% of scored flies had noticeable wing defects or RNAi knockdown lines in which more than 29% of the scored flies had wing defects (see Methods for details).

Using this approach, we identified 15 RNAi knockdown lines (Table 1) and 29 loss-of-function mutant lines (Table 2) with wing abnormalities. In total, 44 unique ion channels that contribute to wing development were identified. The majority of the identified genes (81.8) have not been previously identified as impacting wing development, and 31 of these genes have human orthologs (Table 3). To further examine the candidates of interest, we divided them into six groups based on ion channel type (calcium, sodium, potassium, chloride, ligand-gated cation channel, and other) (Tables 1 and 2). The majority of the ion channels identified in our screen (29.5%) were ligand-gated cation channels, but channels from several categories were represented (Table 4).

**Table 1:** Summary of candidates identified in screen of RNAi knockdowns

Stock ID#	Gene name	Protein Function	% Penetrance Male	% Penetrance Female	% Penetrance Total	Phenotype
**Calcium Channels**						
BL27263	*Stim*	CRAC channel regulator	100	100	100	Wings small and malformed, thick veins, blisters
BL31295	*nan*	Transient receptor potential channel*	84	32	54	L5 incomplete, L5 bifurcation
BL31292	*wtrw*	Transient receptor potential channel*	69	39	53	PCV incomplete
**Sodium Channels**						
BL25847	*rpk*	DEG/epithial sodium channel*	100	85	92	L5 incomplete or bifurcation, L4 bifurcation
BL27088	*ppk25*	DEG/epithial sodium channel*	22	37	30	PCV bifurcation, L5 bifurcation
BL25810	*ppk30*	DEG/epithial sodium channel*	73	30	55	PCV incomplete, L4 & L5 bifurcations, L5 incomplete,
**Potassium Channels**						
VDRC 28430	*Irk1*	Inwardly rectifying K^+^ channel	100	91	95	L5 & L4 bifurcations, loss of ACV, thick veins
VDRC 4341	*Irk2*	Inwardly rectifying K^+^ channel	93	85	89	L5 & L4 bifurcations, loss of ACV, thick veins
VDRC 3886	*Irk3*	Inwardly rectifying K^+^ channel	100	30	47	L5 & L4 bifurcations, loss of ACV, thick veins
**Chloride Channels**						
BL42654	*Best2*	Calcium activated chloride channel*	100	100	100	Wings small and severely malformed
BL39040	*Best3*	Chloride channel*	100	40	66	Small narrow wings (male), missing ACV, L2 bifurcation, PCV incomplete
**Ligand-gated Cation Channels**						
BL62391	*Ir7b*	Ionotropic receptor*	86	97	92	PCV incomplete or bifurcation, L5 bifurcation
BL34678	*Ir76a*	Ionotropic receptor	97	46	74	PCV incomplete, L5 bifurcation
BL53975	*Ir94h*	Ionotropic receptor*	24	29	27	Bristle defects
**Other**						
BL30501	*Inx3*	Gap junction channel	98	38	68	PCV incomplete, L4 bifurcation, L5 bifurcation, L5 incomplete

At least 20 female and 20 male flies were scored for each line.

BL, Bloomington Drosophila Stock Center number, VDRC, Vienna Drosophila Resource Center number.

PCV, posterior cross vein, ACV, anterior cross vein, L, longitudinal vein.

* Function predicted by sequence similarity.

**Table 2:** Summary of candidates identified in screen of loss-of-function mutant lines

Stock ID#	Gene Name	Protein Function	% Penetrance Male	% Penetrance Female	% Penetrance Total	Phenotype
**Calcium Channels**						
BL14156	*stj*	Voltage-gated Ca^2+^ channel*	48	60	54	Bristle defects
BL13682	*SERCA*	Calcium-transporting ATPase*	67	76	40	Ectopic veins, ectopic bristles, pigment defect
BL38067	*brv2*	Calcium channel activity*	20	34	28	PCV bifurcation
BL19957	*inaF-A*; *B*; *C*	Calcium channel regulator	15	50	31	PCV bifurcation
**Sodium Channels**						
BL38075	*ppk*	DEG/epithial sodium channel	42	52	47	PCV bifurcation
BL58557	*ppk17*	DEG/epithial sodium channel*	18	31	25	PCV bifurcation, L2 bifurcation, L3 bifurcation
BL37430	*NaCP60E*	Voltage gated sodium channel	81	100	90	PCV bifurcation, pigment abnormality
BL74	*na*	Sodium leak channel complex component*	37	5	22	Bristle defects, ectopic vein
BL42469	*unc80*	Sodium leak channel complex component*	62	24	43	PCV bifurcation, ectopic vein, pigment defect
BL23397	*unc79*	Sodium leak channel complex regulator*	9	36	23	PCV bifurcation
BL13221	*Teh1*	Sodium channel regulator	70	91	81	Black spots below L5
**Potassium Channels**						
BL59167	*Task6*	Two-pore domain potassium channel	100	100	100	PCV bifurcation
BL59589	*SLO2*	Calcium activated potassium channel	6	37	23	PCV incomplete
BL22837	*Shaker*	Voltage-gated potassium channel	21	85	54	PCV bifurcation
BL37284	*KCNQ*	Voltage-gated potassium channel	20	51	29	PCV bifurcation
**Chloride Channels**						
BL6879	*Best1*	Calcium activated chloride channel	70	100	85	PCV bifurcation
BL1687	*Rdl*	GABA-gated chloride channel	92	95	94	L2 incomplete, ectopic bristles, pigment defect
BL6353	*GluClα*	Glutamate-gated chloride channel	60	100	80	Bristle defects
**Ligand-gated Cation Channels**						
BL44812	*Or47a*	Olfactory receptor	32	51	42	PCV bifurcation
BL56583	*Ir67a*	ionotropic receptor*	80	75	77	PCV bifurcation, L3 bifurcation, thick veins
BL31033	*Ir84a*	Ionotropic receptor	50	34	34	PCV bifurcation, thick veins
BL43017	*Ir92a*	Ionotropic receptor	20	23	22	Bristle defects, abnormal vein pigment
BL25551	*Ir94g*	Ionotropic receptor*	30	8.6	20	Bristle defects, abnormal vein pigment
BL37066	*GluRIIB*	Non-NMDA ionotropic glutamate receptor	31	56	44	PCV bifurcation
BL59216	*mAChR-A*	G-protein coupled acetylcholine receptor	21	56	38	PCV bifurcation, L4 incomplete
BL24880	*nAChRα7*	Nicotinic acetylcholine receptor	42	67	52	PCV bifurcation
BL20783	*nAChRα6*	Nicotinic acetylcholine receptor	26	32	29	PCV bifurcation
BL41424	*nAChRα5*	Nicotinic acetylcholine receptor	15	43	26	PCV bifurcation
**Other**						
BL59187	*CG18549*	Ion channel regulatory protein*	69	97	83	PCV bifurcation

At least 20 female and 20 male flies were scored for each line.

BL, Bloomington Drosophila Stock Center number.

PCV, posterior cross vein, L, longitudinal vein.

* Function predicted by sequence similarity.

**Table 3:** Human orthologs of ion channel candidates identified in screen

*Drosophila melanogaster* Gene	Human Ortholog*
**Calcium Channels**	
*stj*	*CACNA2D3*
*SERCA*	*ATP2A1*
*brv2*	*PKD1L2*
*Stim*	*STIM1*
*nan*	*TRPV6*
*inaF-A;B;C*	none
*wtrw*	none
**Sodium Channels**	
*NaCP60E*	*SCN8A*
*narrow abdomen*	*NALCN*
*unc80*	*UNC80*
*unc79*	*UNC79*
*rpk*	*ASIC2*
*ppk*	*ASIC2*
*ppk25*	*ASIC4*
*ppk30*	*ASIC3*
*ppk17*	none
*Teh1*	none
**Potassium Channels**	
*Task6*	*KCNK9*
*SLO2*	*KCNT1*
*Shaker*	*KCNA1*
*KCNQ*	*KCNQ4*
*Irk1*	*KCNJ2*
*Irk2*	*KCNJ2*
*Irk3*	*KCNJ2*
**Chloride Channels**	
*Best1*	*BEST2*
*Best2*	*BEST4*
*Best3*	*BEST4*
*Rdl*	*GLRA4*
*GluClα*	*GLRA1*
**Ligand-gated Cation Channels**	
*GluRIIB*	*GRIK1*
*mAChR-A*	*CHRM1*
*nAChRα7*	*CHRNA7*
*nAChRα6*	*CHRNA7*
*nAChRα5*	*CHRNA7*
*Or47a*	none
*Ir7b*	none
*Ir67a*	none
*Ir76a*	none
*Ir84a*	none
*Ir92a*	none
*Ir94g*	none
*Ir94h*	none
**Other**	
*CG18549*	*MFSD11*
*Inx3*	none

* Human orthologs were identified using the DRSC Integrative Ortholog Prediction Tool (Version 7.1) (Hu *et al.* 2011). Only human orthologs with a DIOPT score > 2 are shown.

**Table 4:** Number of candidates identified for each ion channel type

Ion Channel Type	Number of Candidates	Percentage of Total Candidates
Ligand-gated cation	13	29.5%
Sodium	10	22.7%
Calcium	7	15.9%
Potassium	7	15.9%
Chloride	5	11.4%
Other	2	4.5%

We found a range in penetrance of defects among the candidates of interest in our screen, with some ion channel disruptions (such as Best2 knockdown) resulting in 100% penetrance of wing defects and other channel disruptions giving much lower penetrance of defects. This variability in penetrance could be because increased expression of other ion channels can compensate for reduced function of one ion channel. For example, when *irk2* is deleted or knocked down with RNAi, *irk1* and *irk3* expression increases (Dahal *et al.* 2012). Each of the ion channels that affected wing morphology was a member of an ion channel family that similarly affects transmembrane potential. It could be that variability of penetrance reflects the differing abilities of ion channels to compensate for other members of the family. Alternatively, the variability in penetrance could be because ion channel disruptions likely impact development by changing the transmembrane potential pattern. Transmembrane potential is regulated by a large number of channels and ions and thus is likely subject to a fairly large amount of biological noise. It has been found that in the nervous system, transmembrane potential often varies due to sources of cellular and molecular noise (Faisal *et al.* 2008). Transmembrane potential is likely subject to the same noise in non-nervous system tissue, leading to the variability in penetrance that we found in the results of our screen.

### The ppk, IR, and Best families are highly represented among the identified ion channels

Among the 44 ion channels that contribute to morphogenesis identified in our screen, several belonged to three gene families: the pickpocket family, the ionotropic receptor family, and the bestrophin family. Five of the identified ion channels (*rpk*, *ppk*, *ppk17*, *ppk25*, and *ppk30*) belong to the pickpocket family. Pickpocket family genes encode Degenerin/epithelial sodium (Na^+^) channels (DEG/ENaCs). Totaling 31 members, the pickpocket family is one of the largest families of ion channel genes in *Drosophila melanogaster*. These channels are non-voltage gated, amiloride-sensitive sodium channels, and some have been characterized as ligand or mechanosensory-gated (Zelle *et al.* 2013). Their functions are not well understood, but they have been implicated in chemosensory and mechanosensory roles, with some members playing roles in pheromone detection required for proper male courtship behavior (Ben-Shahar 2011; Adams *et al.* 1998; Lu *et al.* 2012; Starostina *et al.* 2012). While possible developmental functions of the pickpocket genes in *Drosophila melanogaster* have not been previously investigated, many of the pickpocket genes exhibit changing expression patterns throughout early development, supporting the hypothesis that they may play roles in morphogenesis (Zelle *et al.* 2013). Interestingly, in both *Drosophila melanogaster* and in mammals, DEG/ENaC channels have been recently implicated in neuronal roles, with some studies suggesting that they may directly modulate synaptic processes (Hill and Ben-Shahar 2018; Younger *et al.* 2013). Our results suggest that some members of the pickpocket families may play roles in developmental signaling, further expanding the diverse functions of this family.

Another gene family highly represented in our screen is the Ionotropic Receptor family. Seven of the candidates of interest (*Ir7b*, *Ir67a*, *Ir76a*, *Ir84a*, *Ir92a*, *Ir94g*, and *Ir94h*) belong to the Ionotropic Receptor family, including three (*Ir76a*, *Ir84a*, *Ir92a*) belonging to the Antennal Ionotropic Receptor subfamily and four (*Ir7b*, *Ir67a*, *Ir94g*, *Ir94h*) belonging to the Divergent Ionotropic Receptor subfamily. Ionotropic Receptor family members are similar in sequence to Ionotropic glutamate receptors (iGluRs), but they lack glutamate-interacting residues and are thus thought to be non-responsive to glutamate (Benton *et al.* 2009). These channels are ligand-gated and primarily thought to play chemosensory roles in taste and odor reception (Rytz *et al.* 2013). The Antennal Ionotropic Receptors are mostly expressed in the antennae and are thought to play roles in odor reception while the Divergent Ionotropic Receptors are expressed in gustatory neurons and play roles in taste (Rimal and Lee 2018). These receptors are expressed at low levels during development and in the developing wing disc, and our results suggest that they play roles in morphogenesis of the wing in addition to their chemosensory roles.

Three members of the Bestrophin family, *Best1*, *Best2*, and *Best3*, were found to contribute to wing morphogenesis. Bestrophins are non-voltage gated chloride channels. Interestingly, disruption of *Best2* resulted in the most severe wing defects of all of our candidates of interest. Wing-specific Best2 RNAi expression (using the *MS1096-GAL4* driver) caused the wings to be completely shriveled and malformed (Figure 2D). There is evidence that Best2 may be a calcium activated chloride channel (CaC). Best1 may be both a CaC and a volume regulated anion channel (VRAC) (Chien *et al.* 2006; Chien and Hartzell 2007). Our results indicate that the Bestrophins play a key role in *Drosophila* wing development suggesting that the chloride current is important for correct morphogenesis. Indeed, five chloride channels were identified in our screen (Table 3).

### Multiple genes identified have human orthologs associated with morphological defects

We found several of the ion channels that impact *Drosophila* wing development have human orthologs with mutations that are associated with morphological defects. Three of the ion channels from our screen, Irk1, Irk2, and Irk3 are the *Drosophila* orthologs of Kir2.1, which is a channel associated with Andersen-Tawil syndrome (Tristani-Firouzi *et al.* 2002; Yoon *et al.* 2006b; Yoon *et al.* 2006a). We have previously described the effects of Irk/Kir2.1 disruption on fly and mouse development (Dahal *et al.* 2012; Dahal *et al.* 2017; Belus *et al.* 2018). Here we identify seven additional ion channels that have human orthologs that are associated with morphological defects as part of channelopathies in humans (Table 5). These include *Task6* (*KCNK9*), *Nan* (*TRPV4*), *unc80* (*UNC80*), *narrow abdomen* (*NALCN*), and the nicotinic acetylcholine receptors *nAChRα5*, *nAChRα6*, and *nAChRα7* (*CHRNA7*). Interestingly, the genetic lesions that cause the channelopathies associated with these genes in humans are all loss-of-function mutations (Table 5). The *Drosophila* lines scored in our screen are also loss-of-function or knockdown lines, but it is important to note that human channelopathies usually occur as a result of heterozygous mutations while the majority of the *Drosophila* lines we looked at were homozygous, representing a more severe reduction in ion channel function.

**Table 5:** Human channelopathies with *Drosophila* orthologs identified in screen

*Drosophila* Gene	*Drosophila* Line Screened	Observed wing phenotype	Human Ortholog	Associated Channelopathy	Human channelopathy lesion type	Human morphological phenotypes
*Task6*	Mi(MIC) insertion (BL59167)	PCV bifurcation	*KCNK9*	Birk-Barel Syndrome	Heterozygous missense mutation causing channel loss-of-function (Barel *et al.*, 2008)	Facial dysmorphism
*Nanchung*	Wing-specific RNAi knockdown (MS1096 driver with BL31295)	L5 incomplete, L5 bifurcation	*TRPV4*	*TRPV4* skeletal dysplasias	Variety of heterozygous missense mutations causing channel gain-of-function or loss-of-function (Nilius & Voets 2013)	Wide variety of skeletal dysplasias (see Nilius & Voets, 2013 for phenotype summaries)
*unc80*	Mi(MIC) insertion (BL42469)	PCV bifurcation, ectopic vein, pigment defect	*UNC80*	IHPRF2	Homozygous missense and truncating mutations causing channel loss-of-function (Stray-Pedersen *et al.*, 2016)	Facial dysmorphism, small hands and feet (Stray-Pedersen *et al.*, 2016)
*narrow abdomen*	Hypomorphic allele (BL74)	Bristle defects, ectopic vein	*NALCN*	IHPRF1	Homozygous missense and nonsense mutations causing channel loss-of-function (Al-Sayed *et al.*, 2013)	Facial dysmorphism (Al-Sayed *et al.*, 2013)
*narrow abdomen*	Hypomorphic allele (BL74)	Bristle defects, ectopic vein	*NALCN*	CLIFAHDD	Heterozygous missense mutations in the pore-forming domain, suspected to have a dominant-negative effect (Chong *et al.*, 2015)	Severe facial dysmorphism, limb and digit deformities (Chong *et al.*, 2015)
*nAChRα5*	Mi(MIC) insertion (BL41424)	PCV bifurcation	*CHRNA7*	15q13.3 microdeletion syndrome	Heterozygous or homozygous deletion of *CHRNA7* (Hoppman-Chaney *et al.*, 2013)	Facial dysmorphism (Hoppman-Chaney *et al.*, 2013)
*nAChRα6*	P-element insertion (BL20783)	PCV bifurcation				
*nAChRα7*	Antimorphic allele (BL24880)	PCV bifurcation				

PCV, posterior cross vein, L, longitudinal vein, BL, Bloomington Drosophila Stock Center number.

Twik related acid-sensitive K+ channel 6 (*Task6)* encodes a two-pore-domain potassium channel and is the *Drosophila* ortholog of the human *KCNK9* gene. Heterozygous *KCNK9* loss-of-function mutations in humans cause Birk-Barel syndrome, a channelopathy associated with craniofacial defects including elongated face, downturned eyelids, protruding ears, and cleft palate (Barel *et al.* 2008).

Another channel identified in our screen, *Nanchung* (*Nan*), a transient receptor potential channel, is the *Drosophila* ortholog of *TRPV4*. Both loss-of-function and gain-of-function heterozygous*TRPV4* mutations are associated with high number of skeletal dysplasia disorders that cause skeletal defects such as scoliosis and brachydactyly (shortening of the fingers) (Nilius and Voets 2013).

We found that wing morphogenesis was also affected by the reduced function of *unc80* and *narrow abdomen*, the *Drosophila* orthologs of *UNC80* and *NALCN*, respectively. Together with UNC79, these proteins form a cation channel complex (Lu *et al.* 2010). Loss-of-function homozygous mutations in *NALCN* cause infantile hypotonia with psychomotor retardation and characteristic facies-1 (IHPRF1) and loss-of-function homozygous mutations in *UNC80* cause infantile hypotonia with psychomotor retardation and characteristic facies-2 (IHPRF2) (Bramswig *et al.* 2018; Stray-Pedersen *et al.* 2016; Al-Sayed *et al.* 2013). These are two closely related channelopathies associated with mild dysmorphic facial features (Bramswig *et al.* 2018; Stray-Pedersen *et al.* 2016; Al-Sayed *et al.* 2013). Some heterozygous mutations in *NALCN*, speculated to be dominate-negative mutations, cause congenital contractures of the limbs and face, hypotonia, and developmental delay (CLIFAHDD) (Chong *et al.* 2015). CLIFAHDD is a congenital disorder associated with severe craniofacial defects and limb deformities (Chong *et al.* 2015). In our screen, homozygous loss-of-function mutations in the *Drosophila* orthologs *unc80* and *narrow abdomen* both caused wing defects, indicating that these two proteins may play conserved roles in morphogenesis.

Disrupted function of the nicotinic acetylcholine receptors nAChRα5, nAChRα6, and nAChRα7 were also identified in our screen. These three nicotinic acetylcholine receptors are the *Drosophila* orthologs for the human alpha7 nicotinic acetylcholine receptor (encoded by *CHRNA7*). A 15q13.3 microdeletion syndrome, in which *CHRNA7* and five other genes are deleted, causes facial and digital dysmorphisms (Sharp *et al.* 2008). Single-gene deletions of *CHRNA7* also cause 15q13.3 microdeletion syndrome phenotypes, suggesting that deletion of *CHRNA7* is the cause of the syndrome (Hoppman-Chaney *et al.* 2013). Our screen identified all three of the *Drosophila* orthologs of *CHRNA7* indicating that this nicotinic acetylcholine receptor likely plays a conserved role in development.

### Ion channel compensation effects

While we identified 44 ion channels in our screen, it is likely that our results underestimate the true scope of ion channels involved in wing development. Ion channels are often made up of multiple subunits or have multiple family members that are able to compensate for each other when a single channel is disrupted or deleted. In both developmental and non-developmental contexts (such as in cardiac cells) disruption of a single ion channel can cause upregulation of different ion channels to compensate, masking potential phenotypes (Dahal *et al.* 2012; Rosati and McKinnon 2004). This impact of compensation may be more significant for ion channels that come from large families with many members that could potentially compensate for the loss of one member. It is interesting to note that in the results from our screen, ion channels identified from large families such as the pickpocket family (with 31 members) gave more subtle phenotypes than those from smaller families such as the Bestrophin family (with only four members). This may be a result of ion channel compensation, with other ion channel family members being able to perform the function of the disrupted channels to prevent more severe defects from occurring.

### Potential impacts

To confirm the results of the channels in our screen, more experiments will have to be done using rescues and disruptions in other background phenotypes. However, If conserved developmental roles are found for the channels identified in our screen, this would have important implications in human health as ion channels are one of the top targets of known drugs (Overington *et al.* 2006). We used the drug–gene interaction database (DGIdb, www.dgidb.org) to look for known drugs that act upon the human orthologs of the ion channels identified in our screen (Cotto *et al.* 2018). We found that many of the human orthologs of the ion channels that we identified interact with common general anesthetics such as halothane, sevoflurane, isoflurane, and desflurane. Other ion channels that impact wing morphogenesis in flies interact with anti-hypertension drugs such as amiloride, nilvadipine, verapamil, mibefradil. Another subset of ion channels that we found to impact morphogenesis interact with anti-seizure drugs such as topiramate, phenacemide, ezogabine, zonisamide. If the ion channels identified in our screen have conserved roles in morphogenesis, the use of drugs like these during pregnancy needs to be examined closely. In addition, alcohol is known to act upon Kir channels, human orthologs of Irk1, Irk2, and Irk3, which were identified as modifiers of wing development (Dahal *et al.* 2012; Bates 2013). Furthermore, nicotine acts upon nicotinic acetylcholine receptors, three of which were identified as modifiers of development in our screen (*nAChRα5*, *nAChRα6*, and *nAChRα7*). Our results may help to explain the known effects of maternal smoking on fetal development (Hackshaw *et al.* 2011).

### Conclusion

Overall, our screen identified 44 ion channels that impact morphogenesis of the *Drosophila melanogaster* wing, underscoring the overall importance of ion channels in development. It will be interesting to investigate which specific morphogenic pathways are impacted by the disruption of these channels and the mechanisms by which these ion channels impinge upon these pathways.
